# Molecular Subtypes and Targeted Therapeutic Strategies in Small Cell Lung Cancer: Advances, Challenges, and Future Perspectives

**DOI:** 10.3390/molecules30081731

**Published:** 2025-04-12

**Authors:** Daoyuan Huang, Jingchao Wang, Li Chen, Weiwei Jiang, Hiroyuki Inuzuka, David K. Simon, Wenyi Wei

**Affiliations:** 1 Department of Pathology, Beth Israel Deaconess Medical Center, Harvard Medical School, Boston, MA 02215, USA; 2 Department of Neurology, Beth Israel Deaconess Medical Center, Harvard Medical School, Boston, MA 02215, USA; dsimon1@bidmc.harvard.edu

**Keywords:** SCLC, therapeutic, immune checkpoint, POU2F3, ASCL1, NEUROD1, YAP1, targeted therapies, tumorigenesis

## Abstract

Small cell lung cancer (SCLC) is a highly aggressive malignancy characterized by rapid progression, early metastasis, and high recurrence rates. Historically considered a homogeneous disease, recent multi-omic studies have revealed distinct molecular subtypes driven by lineage-defining transcription factors, including ASCL1, NEUROD1, POU2F3, and YAP1, as well as an inflamed subtype (SCLC-I). These subtypes exhibit unique therapeutic vulnerabilities, thereby paving the way for precision medicine and targeted therapies. Despite recent advances in molecular classification, tumor heterogeneity, plasticity, and therapy resistance continue to hinder clinical success in treating SCLC patients. To this end, novel therapeutic strategies are being explored, including BCL2 inhibitors, DLL3-targeting agents, Aurora kinase inhibitors, PARP inhibitors, and epigenetic modulators. Additionally, immune checkpoint inhibitors (ICIs) show promise, particularly in immune-enriched subtypes of SCLC patients. Hence, a deeper understanding of SCLC subtype characteristics, evolution, and the regulatory mechanisms of subtype-specific transcription factors is crucial for rationally optimizing precision therapy. This knowledge not only facilitates the identification of subtype-specific therapeutic targets, but also provides a foundation for overcoming resistance and developing personalized combination treatment strategies. In the future, the integration of multi-omic data, dynamic molecular monitoring, and precision medicine approaches are expected to further advance the clinical translation of SCLC subtype-specific therapies, ultimately improving patient survival and outcomes.

## 1. Introduction

Small cell lung cancer (SCLC) is a highly aggressive form of neuroendocrine malignancy, accounting for 10–20% of all lung cancer cases, with a strong link to tobacco exposure [1,2,3,4,5]. It is characterized by rapid tumor growth, early metastasis, and a high tumor mutation burden (TMB), leading to a poor prognosis [2,6]. At diagnosis, approximately 70% of patients present with extensive-stage disease (ES-SCLC), and despite high initial response rates to platinum-based chemotherapy, nearly all patients relapse within months. Historically, the five-year survival rate for SCLC has been dismally low (~5%) [7,8,9,10]. Notably, the introduction of immune checkpoint inhibitors (ICIs), such as atezolizumab and durvalumab, has modestly improved outcomes, yet long-term survival rates remain poor, with the five-year survival rate still estimated to be under 10% for SCLC patients [11,12,13].

Unlike non-small cell lung cancer (NSCLC), which has long benefited from well-defined oncogenic drivers and targeted therapies, SCLC has traditionally been considered a single disease entity despite early observations of phenotypic variation in cell lines (e.g., classic vs. variant morphology). While key driver mutations in TP53 and RB1 have been firmly established in the vast majority of SCLC cases, the lack of targetable oncogenes has limited stratified therapeutic approaches [13]. However, recent genomic and multi-omic analyses have revealed significant molecular heterogeneity, leading to the classification of distinct SCLC subtypes largely based on transcription factor dependencies [14,15,16,17,18]. Notably, these subtypes exhibit unique therapeutic vulnerabilities, presenting new opportunities for precision medicine.

Despite these advancements, tumor heterogeneity, subtype plasticity, and lack of a standardized classification continue to hinder clinical translation for effective treatment of SCLC patients. Hence, understanding these molecular subtypes is critical to developing personalized treatment strategies. To this end, this review explores SCLC subtypes, their therapeutic implications, and the challenges of implementing precision medicine approaches, aiming to bridge the gap between basic research and clinical application.

## 2. Molecular Subtypes of SCLC

### 2.1. Historical Perspective and Classification Evolution

Small cell lung cancer (SCLC) was historically classified as a single disease entity, in part due to its near-universal inactivation of the TP53 and RB1 tumor suppressors [2]. However, increasing evidence suggests that SCLC is highly heterogeneous, and early classification attempts sought to delineate subtypes largely based on morphology and neuroendocrine (NE) differentiation. Initially, SCLC was divided into the classical and variant subtypes based on histological characteristics. The classical subtype exhibited high NE differentiation, marked by the expression of biomarkers including L-DOPA decarboxylase, neuro-specific enolase, and brain-type creatine kinase, whereas the variant subtype demonstrated lower NE features, faster proliferation, and greater chemotherapy resistance [19].

**Figure 1:**
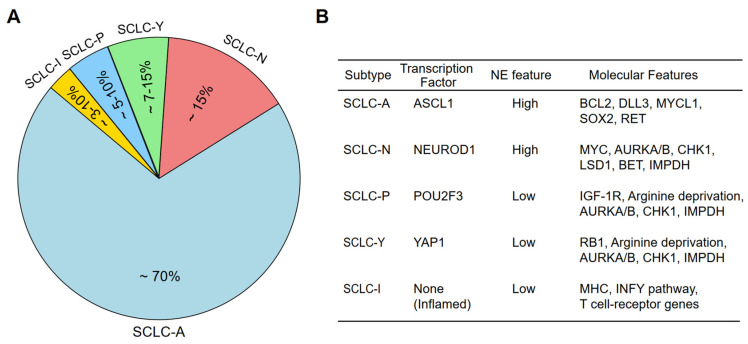
SCLC molecular subtypes and main gene alterations associated with each subtype. (**A**) Relative proportion of the SCLC subtypes among total SCLC cases. (**B**) Main gene alterations present in each of the SCLC subtypes.

Importantly, recent advancements in genomic, transcriptomic, and proteomic analyses have revealed that SCLC consists of distinct molecular subtypes that drive tumor progression and therapeutic responses [16,17,18,20]. This has led to a transcription factor-based classification, identifying four core SCLC subtypes based on the expression of lineage-defining transcription factors (TFs): ASCL1 (SCLC-A), NEUROD1 (SCLC-N), POU2F3 (SCLC-P), and YAP1 (SCLC-Y) [14] (Figure 1A,B). ASCL1 and NEUROD1 define the majority of neuroendocrine-high (NE-high) SCLCs, while YAP1 and POU2F3 mark the non-neuroendocrine (NE-low) subtypes. More recently, multi-omic studies and non-negative matrix factorization (NMF) analyses have identified additional subtypes, including the SCLC-Inflamed (SCLC-I) phenotype, which exhibits immune-related gene expression, as well as new classifications integrating DNA repair, metabolic, and epigenetic dependencies [17,18,21].

### 2.2. Transcription Factor-Based Subtypes of SCLC

Molecular profiling of SCLC tumors and cell lines has identified four major subtypes, each defined by a key transcription factor essential for differentiation and tumor progression [14,22,23,24,25]. In the context of cancer taxonomy, a molecular subtype typically refers to a biologically coherent group of tumors defined by: (1) a dominant transcriptional regulator or signaling axis, (2) relative stability of this signature across disease stages and models, and (3) correlation with distinct biological behaviors or therapeutic vulnerabilities in the clinic. In SCLC, subtypes driven by ASCL1, NEUROD1, and POU2F3 meet these criteria, as each defines a transcriptionally and epigenetically stable state linked to neuroendocrine identity, cell lineage, and drug response. By contrast, candidate subtypes such as SCLC-Y remain under scrutiny in part due to inconsistencies across models and patient samples, as well as their dynamic and often context-dependent expression patterns [18,26]. Hence, establishing clear criteria for what constitutes a true molecular subtype is essential to interpreting data from less established categories such as SCLC-Y. To this end, ASCL1-driven SCLC (SCLC-A) is the most common subtype, accounting for approximately 70% of cases, and is characterized by high ASCL1 expression [26]. Biologically, ASCL1 plays a crucial role in tumor initiation and maintenance by regulating MYCL, SOX2, RET, BCL2, and DLL3, all of which contribute to NE differentiation and tumor survival [23]. This subtype also exhibits relatively high expression of BCL2 and DLL3, thereby making these molecules potential therapeutic targets for the SCLC-A subtype. Importantly, SCLC-A can be further divided into two subtypes: SCLC-Aα, which expresses NKX2-1 and SOX1 and maintains a strong NE phenotype; and SCLC-Aσ, which lacks these markers and may represent a transition state toward NE-low tumors [21].

NEUROD1-driven SCLC (SCLC-N) accounts for ~15% of SCLC cases and is characterized by high NEUROD1 expression, often co-expressed with MYC, which drives rapid tumor proliferation [14,23]. NEUROD1-positive tumors have been associated with an aggressive phenotype and resistance to standard chemotherapy [27]. Furthermore, this subtype exhibits increased expression of Aurora kinase A/B (AURKA/AURKB), making cell cycle inhibition a potential therapeutic approach [23]. Two distinct subtypes have been suggested to exist within SCLC-N: NEv1, which is highly MYC-driven and associated with cell cycle dysregulation; and NEv2, defined by ELF3 and NR0B1 expression, potentially reflecting an alternative differentiation lineage [21,28].

POU2F3-driven SCLC (SCLC-P) represents a non-neuroendocrine (NE-low) subtype and accounts for approximately 7–15% of SCLC cases [29,30]. Notably, POU2F3 functions as a master regulator of tuft cell differentiation and is mutually exclusive with ASCL1 and NEUROD1 expression. Moreover, SOX9, ASCL2, and IGFR1 have been identified as downstream targets of POU2F3 and likely play crucial roles as selective dependencies in POU2F3-driven tumors [29]. SCLC-P tumors lack classic NE markers, but rely on IGF1R signaling, therefore making them potential candidates for IGF1R-targeted therapies [29]. SCLC-P tumors also exhibit deficiencies in DNA repair pathways, suggesting that they may be more sensitive to DNA-damaging agents such as PARP inhibitors [23].

The classification of YAP1-driven small cell lung cancer (SCLC-Y) remains highly debated, in part due to conflicting findings from various research models and clinical datasets [16,26,31,32]. Unlike the well-established subtypes defined by ASCL1 (SCLC-A), NEUROD1 (SCLC-N), and POU2F3 (SCLC-P), the existence of a distinct YAP1-driven subtype has not been consistently confirmed across studies. While some analyses identify YAP1 expression in a subset of non-neuroendocrine (NE-low) SCLC tumors, others suggest that its presence is rather transient, potentially representing a late-stage adaptation rather than a fundamental stable subtype [22,33].

Initial studies suggested that YAP1 expression was restricted to a subset of NE-low SCLC tumors, but comprehensive profiling has yielded mixed results. Some reports identify YAP1 expression in 2.8–10% of clinical SCLC samples, while others do not detect significant YAP1 levels in genetically engineered mouse models (GEMMs), patient-derived xenografts (CDXs), or human tumors [14,16,26,31,34]. A meta-analysis of 1000 neuroendocrine carcinomas, including SCLC, supports the presence of a YAP1-expressing subtype and even suggests an additional classification based on HNF4A expression, pointing to a potential extra-thoracic small cell carcinoma variant [35]. Interestingly, a key regulatory mechanism influencing YAP1 expression in SCLC involves RB1. To this end, the near-universal loss of RB1 in SCLC leads to E2F7-mediated suppression of YAP1, suggesting an inverse relationship between RB1 genetic status and YAP1 expression [36,37]. Consistent with this notion, rare RB1-proficient SCLC tumors exhibit relatively lower neuroendocrine marker expression and are enriched for YAP1, implying that YAP1-driven tumors may arise in cases where RB1 loss is incomplete or functionally compensated [38]. Further complicating SCLC-Y classification, some previously identified YAP1-positive SCLC cell lines have been reclassified as SMARCA4-mutant undifferentiated tumors (SMARCA4-UT), a distinct entity with aggressive biology and poor differentiation [37]. While both SCLC and SMARCA4-UT share high proliferative capacity (Ki67 positivity of 80–100%) and TP53 mutations, SMARCA4-UT tumors differ in their low TTF1 expression, lack of neuroendocrine markers, and high SMARCA4 mutation burden [37,39,40].

In early-stage SCLC GEMMs, YAP1 expression is rarely detected, as these models are generated based on dual Rb1/Trp53 loss [41,42]. However, in GEMMs incorporating oncogenic Myc activation, YAP1-positive tumors emerge in late-stage disease, with single-cell analyses indicating that these NE-low, YAP1-expressing tumors may originate from either SCLC-A or SCLC-N lineages [22,33]. Additionally, YAP1 expression is more frequently detected in recurrent or treatment-resistant SCLC tumors, therefore supporting its critical role in tumor progression and plasticity [22,33,43,44]. Interestingly, low and heterogeneous YAP1 expression is often observed alongside other lineage-defining transcription factors, making it challenging to define YAP1 as an exclusive molecular marker for SCLC [18,26]. Unlike in non-small cell lung cancer (NSCLC) cases, where YAP1 is strongly expressed and contributes to oncogenic processes, YAP1 in SCLC appears to correlate with limited-stage disease, an inflamed tumor microenvironment, and improved prognosis [26,44]. This raises the possibility that YAP1 expression may mark a dynamic transition state rather than a stable, lineage-defined SCLC subtype, which warrants future in-depth studies.

The co-expression of YAP1 with other transcription factors further complicates its role as a standalone lineage marker. Future research should leverage advanced genomic, epigenomic, and single-cell transcriptomic approaches to determine whether YAP1-driven tumors constitute a true molecular subtype or merely reflect a transient state influenced by tumor plasticity. Integrating longitudinal tumor evolution studies with treatment response data may help clarify whether targeting YAP1-associated pathways could provide therapeutic benefits for a subset of SCLC patients. In conclusion, while YAP1 expression plays a role in SCLC progression and therapy resistance, its classification as a distinct SCLC subtype remains debatable at the current stage. Hence, further investigations are necessary to establish whether YAP1-driven tumors represent a unique biological entity or a dynamic adaptation to oncogenic and environmental pressures.

### 2.3. Emerging Subtypes and Multi-Omic Classifications

Beyond the classical transcription factor-based classification, additional SCLC subtypes have been proposed based on emerging multi-omic studies. For example, ATOH1-driven SCLC is a newly proposed subtype identified in cell-derived xenograft (CDX) models that lack ASCL1, NEUROD1, and POU2F3 expression [16]. While initially considered an independent entity, ATOH1-expressing tumors may represent a subset of SCLC-N, indicating further complexity within the NEUROD1-driven lineage [16,45]. Another emerging subtype is SCLC-Inflamed (SCLC-I), which is characterized by high immune infiltration and elevated immune-related gene expression [18] (Figure 1A,B). SCLC-I tumors are characterized by a relative lack of MHC class I/II repression, along with elevated PD-L1 and interferon-gamma (IFN-γ) signaling components, suggesting enhanced antigen presentation and potential sensitivity to immune checkpoint inhibitors (ICIs) [18,34,46,47,48]. Further analyses have also identified two distinct subtypes within SCLC-I: NE-SCLC-I, which retains some NE characteristics and is associated with higher T-effector cell infiltration and potential ICI responsiveness; and Non-NE-SCLC-I, which exhibits POU2F3 expression, with limited benefit from immunotherapy [49].

In addition to these immune-enriched subtypes, recent non-negative matrix factorization (NMF) analyses have further expanded SCLC classification by integrating transcriptomic, proteomic, and phospho-proteomic signatures [17]. These analyses have identified four novel SCLC subtypes: NMF1, which is enriched in DNA damage repair and cell cycle pathways and may benefit from PARP inhibitors; NMF2, characterized by a high tumor mutational burden (TMB) and DLL3 expression, suggesting responsiveness to DLL3-targeted agents and immune checkpoint blockade; NMF3, which exhibits strong RTK signaling activity and may be susceptible to RTK inhibitors; and NMF4, which is linked to RNA metabolism and MYC pathway activation, indicating potential sensitivity to Aurora kinase inhibitors [36].

Despite these advancements, significant challenges remain in applying molecular classification to clinical practice. Mechanistically, intratumoral heterogeneity is a major obstacle, as many SCLC tumors exhibit overlapping expression of multiple subtype markers, complicating treatment selection [32]. Single-cell RNA sequencing (scRNA-seq) analyses have revealed that up to 37% of SCLC tumors co-express ASCL1 and NEUROD1, suggesting that these subtypes are not always mutually exclusive [26]. Moreover, a small fraction of tumors (~6.3%) remain negative for all four major transcription factor markers, further complicating subtype-specific treatment strategies [21,32,38]. The dynamic evolution of SCLC under therapeutic pressure poses another challenge, as tumors can transition between subtypes in response to treatment.

### 2.4. Challenges in SCLC Subtyping

The lack of a standardized classification system presents a major hurdle in translating subtype-based findings into clinical applications. Different classification methods, ranging from RNA-sequencing and methylation profiling to immunohistochemistry (IHC), have resulted in inconsistencies in subtype definitions. Additionally, limited access to tumor biopsies in SCLC patients complicates real-time molecular classification. Liquid biopsy-based approaches, including circulating tumor DNA (ctDNA) analysis, have emerged as potential tools for non-invasive subtype classification and treatment monitoring [50,51]. Recent studies have demonstrated that SCLC subtypes can also be identified through ctDNA methylation signatures, thereby offering a promising avenue for clinical implementation [50].

Overall, the molecular classification of SCLC has shifted the paradigm in how this aggressive cancer type is understood and treated. While transcription factor-based subtypes provide a foundational framework, emerging multi-omic and immunogenomic insights are further reshaping our understanding of SCLC heterogeneity. As new therapeutic targets emerge, integrating molecular subtyping into clinical decision-making will be critical for improving patient outcomes and advancing the field of precision oncology in SCLC.

## 3. Upstream Regulation Mechanism of Lineage-Defining Transcription Factors

Given the critical role of lineage-defining transcription factors in driving SCLC subtypes, understanding their regulatory mechanisms is essential for identifying strategies to downregulate their expression, offering potential therapeutic avenues for targeted SCLC treatment.

### 3.1. Regulation of ASCL1

It has been reported that HUWE1 functions as an E3 ubiquitin ligase of ASCL1 to promote its ubiquitination and subsequent degradation [52]. On the other hand, TCF3 stabilizes ASCL1 by promoting its multi-site phosphorylation via the CDK2–CyclinA2 kinase complex, preventing its degradation [53]. During mitosis, TCF3 dissociates from ASCL1, leading to its degradation, while chemotherapy significantly reduces ASCL1 transcription, enhancing SCLC cell sensitivity to treatment [53]. In contrast, knockdown of *USP8* promotes ASCL1 ubiquitination and its degradation in hBMSCs [54], indicating that USP8 inhibitors may play a candidate role in SCLC-A tumorigenesis.

### 3.2. Regulation of NEUROD1

Recent transcriptomic and epigenetic analyses have revealed that NEUROD1 expression is tightly regulated by specific chromatin remodeling factors and histone modifications. SMARCA4, a key component of the SWI/SNF chromatin remodeling complex, plays a crucial role in SCLC state plasticity by modulating the transcription of NEUROD1 and other neuroendocrine lineage-defining factors [55]. Loss of SMARCA4 function disrupts NEUROD1 expression, leading to changes in tumor differentiation and resistance to therapy. Additionally, histone methylation regulators such as EZH2 have been implicated in silencing NEUROD1 expression in certain contexts [20,56], whereas LSD1 (KDM1A) has been shown to enhance its transcriptional activity [57]. On the other hand, FMRP negatively regulates NEUROD1 expression by binding to its mRNA, thereby modulating glutamatergic neuronal differentiation [58].

### 3.3. Regulation of POU2F3

POU2F3 is a master regulator of tuft cell identity in a variant form of SCLC that lacks neuroendocrine features, functioning as a key lineage determinant. Its expression is regulated by lineage-specific transcription factors SOX9 and ASCL2 and is associated with IGF1R signaling, highlighting potential therapeutic targets for SCLC-P tumors [29]. In addition, POU2F3 regulates tuft cell identity in tuft-cell-like SCLC, largely through its interaction with the coactivators OCA-T1 (POU2AF2) and OCA-T2 (POU2AF3), forming a tertiary transcriptional complex essential for lineage specification. More importantly, disrupting this POU2F3–OCA-T axis eliminates tuft cells, highlighting a critical molecular vulnerability in SCLC-P, which could serve as a potential therapeutic target [30]. Recent studies have identified mSWI/SNF (mammalian switch/sucrose non-fermentable) chromatin remodeling complexes as key regulators of POU2F3 expression in tuft cell-like small cell lung cancer (SCLC-P), highlighting a crucial lineage-specific dependency [59,60]. Mechanistically, the mSWI/SNF complex maintains chromatin accessibility at gene loci essential for the POU2F3 regulatory network, thereby sustaining POU2F3 expression and downstream signaling in SCLC-P tumors [59,60]. This dependency is unique to POU2F3-positive SCLCs, highlighting it as a potential therapeutic target. These findings, therefore establish mSWI/SNF inhibition as a promising therapeutic strategy for SCLC-P by selectively disrupting its lineage-defining transcriptional program. Moreover, POU2AF2 (OCA-T1) functions as a critical co-activator of POU2F3, regulating tuft cell identity in SCLC-P by modulating enhancer activity and maintaining chromatin accessibility via interaction with the SWI/SNF complex. Loss of POU2AF2 disrupts 3D chromatin structure, leading to transcriptional reprogramming and PTEN-dependent cell growth suppression. Targeting the POU2AF2–SWI/SNF axis presents a promising therapeutic strategy for inhibiting POU2F3-driven tumor progression in SCLC-P [61].

Overall, ASCL1, NEUROD1, and POU2F3 are the three most well-established lineage-defining transcription factors in SCLC. Here, we focus solely on their upstream regulatory mechanisms (Table 1). While some regulatory pathways have been identified, particularly the ubiquitination-mediated degradation of ASCL1, the contribution of the post-translational regulation of these transcription factors as well as their physiological roles in SCLC pathogenesis remains largely unknown. Given the critical role of these factors in SCLC, further research is urgently needed to elucidate their upstream regulatory networks, especially at the protein level. Unraveling these mechanisms will provide essential theoretical foundations for the development of selective therapeutic strategies targeting SCLC subtypes.

## 4. Targeted Therapeutic Strategies for SCLC Subtypes

The recent advancements in the molecular characterization of small cell lung cancer (SCLC) have led to the identification of distinct subtypes, each exhibiting unique therapeutic vulnerabilities. These findings have facilitated the development of subtype-specific targeted therapies, including small-molecule inhibitors, immunotherapy, and combination strategies aimed at overcoming resistance. This section discusses the current and emerging therapeutic strategies tailored to different SCLC subtypes (Table 2), highlighting their molecular dependencies and potential clinical applications.

### 4.1. ASCL1-Driven SCLC (SCLC-A)

SCLC-A, accounting for approximately 70% of SCLC cases, is driven by ASCL1, a master regulator of neuroendocrine (NE) differentiation [23,35]. This subtype is characterized by high expression of BCL2, DLL3, SOX2, RET, and MYCL1 [23]. One of the most promising therapeutic targets in SCLC-A is BCL2, an anti-apoptotic protein directly regulated by ASCL1. BCL2 inhibitors, such as venetoclax, have shown significant preclinical efficacy in SCLC models with high BCL2 expression [63,64,65,66]. Additionally, *DLL3*, another ASCL1-regulated gene, has emerged as a target for antibody-drug conjugates (ADCs) [67]. While rovalpituzumab tesirine (Rova-T) failed in clinical trials, the DLL3-targeting bispecific T-cell engager tarlatamab has recently gained accelerated FDA approval, showing promise in SCLC treatment [67,68].

Epigenetic regulators, including LSD1, EZH2, and KDM5A, have been implicated in the maintenance of ASCL1 expression [62,65,69]. Hence, LSD1 inhibitors (e.g., ORY-1001) and histone deacetylase (HDAC) inhibitors such as pracinostat have demonstrated potential in preclinical models by downregulating ASCL1 and suppressing tumor growth [41,70,71]. Additionally, ASCL1-driven SCLC exhibits ATR pathway dependency, and ATR inhibitors have shown selective efficacy in preclinical studies [72]. In the SCLC-A subtype, Schlafen family member 11 (SLFN11) exhibits a bimodal expression pattern [73]. High SLFN11 levels have been associated with enhanced sensitivity to cisplatin and poly (ADP-ribose) polymerase (PARP) inhibitors, such as olaparib, confirming its potential as a predictive biomarker for treatment response [18,23]. Additionally, carcinoembryonic antigen-related cell adhesion molecule 5 (CEACAM5) is highly expressed in ASCL1-driven SCLC and represents a promising therapeutic target, with labetuzumab govitecan being a potential targeting agent [18]. Moreover, Lurbinectedin, a recently approved drug, has been shown to suppress the expression of ASCL1-related genes, including MYC, AURKA, and BCL2, and may offer greater benefits in this subtype [74,75].

### 4.2. NEUROD1-Driven SCLC (SCLC-N)

SCLC-N, comprising approximately 15% of cases, is characterized by NEUROD1 activation and frequent MYC amplification, leading to rapid proliferation and chemoresistance [14,23]. MYC is a key oncogenic driver in SCLC-N, making MYC-targeting strategies crucial for therapeutic intervention. While direct MYC inhibition remains challenging, novel agents such as OMO-103, which disrupts MYC-MAX dimerization, are currently in early clinical development (NCT04808362) [76]. Preclinical and clinical studies suggest that MYC-high SCLC tumors exhibit selective sensitivity to Aurora kinase A/B (AURKA/AURKB) inhibitors, such as alisertib or barasertib, which impair MYC-driven tumor growth [22,77,78]. Moreover, in ASCL1-low SCLC, inosine monophosphate dehydrogenase-1 and -2 (IMPDH1 and IMPDH2) are selectively expressed as downstream targets of MYC [79]. As such, targeting IMPDH has shown enhanced suppression of SCLC growth, highlighting its therapeutic potential. Likewise, MYC-driven SCLC demonstrates increased sensitivity to arginine deprivation and mTOR inhibition, particularly in combination with checkpoint kinase 1 (CHK1) inhibitors [43,77]. These findings underscore the promise of targeted inhibitors for treating NEUROD1-driven and ASCL1-low SCLC subtypes with MYC overexpression.

NEUROD1 also functions as a coactivator of BET proteins (e.g., BRD4), therefore making BET inhibitors (CPI-0610, JQ1) a promising therapeutic option [80]. Additionally, KSR1, a key mediator of cisplatin resistance, has been identified as a potential target in SCLC-N [81]. High expression of somatostatin receptor 2 (SSTR2) has been detected in NEUROD1-positive tumors, suggesting that somatostatin analogs may hold therapeutic potential [82].

### 4.3. POU2F3-Driven SCLC (SCLC-P)

SCLC-P, found in approximately 7–15% of cases, is defined by POU2F3 expression and a lack of NE features [14,35]. This subtype is characterized by distinct metabolic dependencies and increased reliance on IGF1R signaling [29]. Preclinical CRISPR screening has identified IGF1R inhibitors (linsitinib) as a promising treatment strategy [29]. Additionally, SCLC-P tumors exhibit increased sensitivity to PARP inhibitors and antifolate agents [18]. Recent studies found that the mSWI/SNF chromatin remodeling complex is essential for maintaining POU2F3 expression and tuft cell identity in POU2F3-driven small cell lung cancer (SCLC-P) [59,60]. As such, pharmacological inhibition of SWI/SNF ATPases (e.g., SMARCA4/2 inhibitors and BRD9 degraders) disrupts chromatin accessibility at POU2F3 regulatory loci, leading to transcriptional reprogramming and impaired tumor growth [59]. Loss of POU2AF2, a co-activator of POU2F3, similarly alters chromatin structure and gene expression by redistributing mSWI/SNF occupancy, revealing an intrinsic dependency on this complex [61]. Thus, SWI/SNF ATPase inhibitors represent a promising therapeutic strategy for SCLC-P, selectively targeting its epigenetic vulnerabilities and offering a potential approach to restrict tumor progression. Given the distinct biology of POU2F3-driven tumors, further investigation into their metabolic vulnerabilities is warranted.

### 4.4. Inflamed SCLC (SCLC-I) and Immunotherapy Approaches

SCLC-I represents an immune-inflamed phenotype, characterized by high MHC class I/II expression, IFN-γ signaling, and immune checkpoint molecules [18]. This subtype exhibits enhanced CD8+ T-cell infiltration and increased antigen presentation, making it particularly susceptible to immune checkpoint inhibitors (ICIs) [47,83]. In the CASPIAN phase III trial, MHC-I/II-high (median OS: 23.6 vs. 10.4 months) and CD8A-high (median OS: 25.1 vs. 10 months) gene signatures were associated with the greatest survival benefit from ICI therapy [84].

In addition to ICIs, BTK inhibitors (ibrutinib) and HDAC inhibitors (pracinostat) have been proposed as potential agents to enhance immune response in SCLC-I tumors [18]. Furthermore, HDAC inhibitors have been identified in pre-clinical studies as having potential protective effects in PD models [85,86,87]. Future studies should explore combinatorial strategies integrating immunotherapy with targeted epigenetic modifiers to combat human diseases such as SCLC and Parkinson’s disease.

### 4.5. Therapeutic Features Beyond Classical Subtypes

In addition to the four major transcription factor-defined SCLC subtypes (SCLC-A, -N, -P, and -I), recent studies have identified a set of molecular features that do not necessarily constitute new subtypes but represent cross-cutting biological states or treatment-associated phenotypes. These features, including YAP1 activation, RB1-intact profiles, and molecular clusters identified through unsupervised algorithms such as non-negative matrix factorization (NMF), provide further insight into the heterogeneity and plasticity of SCLC. These phenotypes have potential therapeutic implications, particularly in the context of relapse, resistance, and non-neuroendocrine transformation.

#### 4.5.1. YAP1 Activation in Relapsed or Therapy-Resistant SCLC

While YAP1 has been previously proposed as the defining feature of a distinct SCLC subtype (SCLC-Y), accumulating evidence suggests that YAP1 activation is more accurately described as a therapy-induced or resistance-associated phenotype rather than a de novo molecular subtype. YAP1, a transcriptional coactivator of the Hippo signaling pathway, is frequently upregulated in non-neuroendocrine and mesenchymal-like SCLC tumors, particularly following chemotherapy or immune checkpoint blockade [88,89]. This phenotype is characterized by epithelial-mesenchymal transition (EMT)-like traits, reduced neuroendocrine marker expression, and pronounced resistance to standard therapies. Preclinical models have shown that receptor tyrosine kinase (RTK) inhibitors, particularly those targeting EGFR and FGFR, may suppress growth in YAP1-high SCLC cells [26,37,90]. Additionally, Bruton’s tyrosine kinase (BTK) is highly expressed in this context, and BTK inhibitors such as ibrutinib have demonstrated activity in YAP1-positive preclinical models [91].

While these findings suggest potential therapeutic strategies for YAP1-driven resistance, the clinical utility of directly targeting YAP1 remains under investigation. YAP1 expression may also intersect with neurodegenerative pathways, such as those implicated in Alzheimer’s and Parkinson’s disease, although these associations currently remain within the preclinical realm [92].

#### 4.5.2. RB1-Intact Phenotype in SCLC: Biology or Artifact?

Most SCLC tumors exhibit bi-allelic inactivation of RB1, a hallmark genetic alteration that underpins loss of cell cycle control [2,93]. However, a minority of tumors have been reported to retain RB1 expression or function, raising the possibility of a distinct RB1-intact subset. This interpretation, however, is contentious. Many in the field argue that these cases may represent phenocopies of RB1 loss—through mechanisms such as CDK4/6 hyperactivation, p16INK4A loss, or functional inactivation via upstream deregulation—rather than truly RB1-proficient SCLC [38,94].

Nonetheless, RB1-intact SCLC cells have demonstrated augmented sensitivity to CDK4/6 inhibitors (e.g., palbociclib, abemaciclib) in both preclinical and early translational studies [38]. These observations support the rationale for therapeutic stratification based on RB1 status. To resolve this controversy, future studies must incorporate protein-level confirmation of RB1 functionality, transcriptomic validation, and integration of CDK pathway biomarkers. Given the evolving understanding of RB1 biology in SCLC, targeting CDK4/6 in a subset of tumors with apparent RB1 function remains a promising but investigational approach.

#### 4.5.3. Molecular Patterns Identified by NMF Clustering

Unsupervised clustering algorithms, including non-negative matrix factorization (NMF), have been applied to transcriptomic datasets to uncover distinct molecular programs in SCLC. While some of these clusters recapitulate known subtypes (such as ASCL1, NEUROD1, and POU2F3-driven tumors), others reflect unique pathway enrichments and therapeutic vulnerabilities that cut across traditional subtype boundaries [17]. For instance, NMF1 is enriched in DNA damage repair and cell cycle-related genes, indicating potential sensitivity to PARP and ATR inhibitors. NMF2 exhibits high tumor mutational burden (TMB) alongside elevated DLL3 expression, suggesting that it may respond to DLL3-targeted antibody-drug conjugates (ADCs) and immune checkpoint inhibitors. In contrast, NMF3 is defined by prominent receptor tyrosine kinase (RTK) activity, implicating therapeutic vulnerability to EGFR or FGFR inhibitors. Finally, NMF4 displays a MYC-like transcriptional profile with enhanced RNA metabolism, rendering it particularly susceptible to Aurora kinase inhibition [17].

It should be noted that clustering methods such as NMF have also been employed in other studies (e.g., Gay et al.) [18], often supporting the stability of the classical four-subtype framework. As such, these clusters are best viewed as molecular overlays or phenotypic gradients, rather than definitive new subtypes. Nonetheless, they provide useful granularity for biomarker-guided treatment selection, particularly in the context of combination strategies and resistance mechanisms.

## 5. Challenges in Targeting SCLC Subtypes

Despite significant advancements in molecular classification and targeted therapy development, several challenges persist in translating these findings into clinical practice. The complexity of SCLC, driven by tumor heterogeneity, dynamic clonal evolution, resistance mechanisms, and the limitations of biomarker-driven approaches, continues to hinder treatment success (Table 3). Addressing these barriers is crucial to optimizing precision medicine strategies for SCLC.

### 5.1. Heterogeneity and Tumor Evolution

Therapeutic resistance in small cell lung cancer (SCLC) is profoundly influenced by its complex intratumoral heterogeneity (ITH) and dynamic cell state plasticity. Increasing evidence suggests that SCLC is not a static, uniform disease, but rather a constellation of molecular subpopulations that can coexist and evolve over time, particularly under therapeutic pressure.

Pioneering efforts employing single-cell and CTC-derived models have revealed that resistant tumors exhibit divergent transcriptional states, including upregulation of stress response pathways, partial epithelial-mesenchymal transition (EMT), and altered expression of therapeutic targets. These adaptive changes do not occur in isolation but reflect the expansion or selection of transcriptionally distinct subclones following treatment exposure. Longitudinal analyses of CTCs have further confirmed that such heterogeneity increases post-relapse, reinforcing the value of noninvasive approaches to monitor tumor evolution in real time [95].

Beyond cellular diversity, recent advances in spatial transcriptomics and proteomics have exposed another critical layer of complexity: regional heterogeneity within tumors. High-resolution spatial profiling studies have demonstrated that SCLC tumors harbor distinct molecular identities and immune microenvironments across histologically adjacent regions, even in treatment-naïve cases [96]. Notably, neuroendocrine differentiation markers, immune cell localization, and signaling pathway activity vary significantly within the same tumor, complicating biomarker interpretation and therapeutic targeting. Spatially confined immune activation has been observed in low-neuroendocrine regions, suggesting that cell lineage plasticity may shape immune visibility and response potential.

Moreover, spatially defined gene expression patterns have been linked to patient outcomes, and integrative analyses have led to the development of ITH-based prognostic classifiers such as ITHtyper, which stratifies tumors based on regional heterogeneity scores [97]. These efforts collectively underscore that resistance in SCLC is rarely driven by a single dominant mechanism. Instead, multiple coexisting subpopulations, each with distinct vulnerabilities, often emerge following therapy. Thus, rationally designed combination treatments, implemented early and guided by longitudinal and spatially resolved profiling, may offer the best chance to suppress the development of heterogeneous, treatment-refractory clones.

Clonal evolution further exacerbates the challenge of therapeutic resistance [98,99]. SCLC is notorious for its ability to rapidly adapt to treatment, with recurrent tumors often displaying distinct molecular features compared to the primary tumor. This suggests that, while initial responses to therapy may be robust, surviving tumor cells may acquire mutations or epigenetic changes that drive resistance. To counteract this, longitudinal monitoring of tumor evolution through liquid biopsy techniques and single-cell sequencing will be essential to guide real-time treatment adjustments.

### 5.2. Challenges in Biomarker-Driven Therapy

The success of targeted therapies depends on the ability to accurately stratify patients based on molecular biomarkers. However, SCLC lacks well-established, reliable biomarkers, making patient selection for targeted treatments challenging. While transcription factor expression (e.g., ASCL1, NEUROD1, POU2F3, and YAP1) has been used to classify SCLC subtypes, these markers alone may not be sufficient to predict treatment response. Additional molecular features, such as MYC amplification, SLFN11 expression, DLL3 levels, and immune-related signatures, need to be incorporated into patient selection strategies.

One potential solution lies in the use of liquid biopsy-based biomarkers, including circulating tumor DNA (ctDNA), circulating tumor cells (CTCs), and exosomal RNA profiling [50,51,100]. These non-invasive approaches allow for real-time assessment of tumor evolution and therapy responses without the need for repeated invasive biopsies. Additionally, the integration of single-cell sequencing technologies could provide deeper insights into tumor heterogeneity, identifying subpopulations of resistant clones before they become dominant. Moving forward, the validation of robust biomarkers through large-scale clinical trials will be critical for refining precision medicine approaches in SCLC.

### 5.3. Resistance Mechanisms and Combination Approaches

Resistance to targeted therapies is a well-documented challenge in SCLC, often arising through adaptive survival mechanisms. One key contributor to therapeutic resistance is epigenetic reprogramming, which allows tumor cells to evade drug-induced cell death by transitioning to a different molecular phenotype. For instance, epigenetic plasticity has been implicated in the loss of neuroendocrine features in SCLC, leading to increased resistance to chemotherapy and immune checkpoint inhibitors (ICIs) [101,102,103].

Given the rapid adaptability of SCLC, monotherapy strategies are unlikely to produce durable responses. Instead, combination approaches that target multiple vulnerabilities simultaneously are gaining traction. Metabolic inhibitors targeting vulnerabilities such as arginine deprivation and mTOR signaling could be combined with DNA-damaging agents to enhance treatment efficacy [104]. The use of synthetic lethality approaches, where two co-existing molecular defects are simultaneously targeted, may also provide a novel route to overcoming resistance [105,106,107]. For instance, the combination of PARP inhibitors with ATR or CHK1 inhibitors has demonstrated promising preclinical activity in high-replication stress SCLC subtypes [108].

To improve treatment durability, future research should focus on identifying rational combination strategies tailored to specific SCLC subtypes, integrating knowledge from genomic, proteomic, and metabolic profiling.

### 5.4. Mixed Histology and Transformed SCLC: Distinct Clinical Entities

In addition to the canonical subtypes and resistance-associated phenotypes, mixed histology tumors and transformed SCLC represent distinct clinical entities with implications for diagnosis, treatment, and prognosis.

Mixed histology SCLC, such as tumors exhibiting components of large-cell neuroendocrine carcinoma (LCNEC) or non-small cell lung cancer (NSCLC), are increasingly recognized in both surgical specimens and small biopsies [109,110,111,112]. These tumors often display discordant morphological and immunohistochemical features, harboring genetic alterations characteristic of both SCLC (e.g., TP53, RB1 loss) and NSCLC (e.g., KRAS, STK11 mutations) [113]. Their biological behavior and treatment response remain heterogeneous, and there is currently no standardized therapeutic approach. Some studies suggest that the dominant histologic or molecular component may guide treatment choice, but prospective validation is lacking.

On the other hand, transformed SCLC typically arises as a resistance mechanism in EGFR-mutant or ALK-rearranged lung adenocarcinoma following tyrosine kinase inhibitor (TKI) therapy [114,115,116,117,118]. These transformed tumors often acquire canonical SCLC features—TP53 and RB1 co-inactivation, small cell morphology, and neuroendocrine differentiation—while retaining the original oncogenic driver. Transformed SCLC tends to be highly resistant to chemotherapy and immune checkpoint inhibitors, with limited responses reported in clinical series [119,120]. Their emergence underscores the plasticity of lung adenocarcinoma and the urgent need for molecular re-biopsy at progression to guide therapy.

Both entities challenge the classical paradigm of SCLC as a uniformly chemosensitive, treatment-naïve disease. Future research should focus on developing diagnostic criteria, lineage-tracing tools, and tailored treatment strategies for these hybrids and transformed forms.

## 6. Conclusions and Future Perspectives

As precision medicine continues to evolve, novel therapeutic strategies are being developed to address the challenges of small cell lung cancer (SCLC). These strategies include next-generation targeted therapies, AI-driven precision oncology, and adaptive clinical trial designs, all aimed at improving patient outcomes through personalized treatment approaches. CRISPR-based gene editing technology and the recently developed DUBTAC technology hold promise for restoring tumor suppressors like TP53 and RB1, while functional genomics screens have identified new druggable targets, thereby paving the way for future interventions [121,122,123,124]. RNA-targeting therapies, including RNA interference (RNAi) and antisense oligonucleotides (ASOs), may offer an innovative means of modulating undruggable transcription factors such as ASCL1, NEUROD1, POU2F3, and YAP1, thereby expanding potential treatment options.

Moreover, AI-driven models are revolutionizing precision oncology by integrating multi-omic data—genomic, transcriptomic, proteomic, and metabolomic—to optimize subtype classification and predict treatment responses. Machine learning can refine therapy selection by identifying effective drug combinations tailored to specific molecular profiles, enabling more precise and adaptive treatment strategies.

Adaptive trial designs are emerging as a crucial approach for evaluating targeted therapies in SCLC. Basket trials, which classify patients based on molecular markers rather than histology, allow for efficient testing of subtype-specific drugs. For instance, Aurora kinase inhibitors could be evaluated across multiple MYC-amplified cancers, including SCLC. Additionally, liquid biopsy-based real-time monitoring of circulating tumor DNA (ctDNA) and transcriptomic changes enables dynamic treatment adjustments, addressing resistance mechanisms as they arise.

Beyond its intrinsic molecular complexity, SCLC exhibits unexpected biological links with neurodegenerative diseases, particularly the onset of Parkinson’s disease (PD) [125,126]. To this end, epidemiological studies suggest an inverse correlation between SCLC and PD, implying potential protective or compensatory mechanisms that influence tumorigenesis and neurodegeneration [125]. Mechanistically, PINK1 overexpression in SCLC contrasts with its loss-of-function role in PD, underscoring mitochondrial stress as a key determinant of cellular fate in both diseases [127]. Additionally, transcription factors such as ASCL1 and NEUROD1, which are crucial for SCLC progression, also play fundamental roles in neuronal differentiation [23,27,128,129,130], therefore suggesting a shared molecular framework in both tumorigenesis and neurodevelopmental disorders. From a clinical standpoint, prolonged chemotherapy in SCLC has been linked to neurological complications, including cognitive decline and parkinsonism-like symptoms [131,132,133]. In this regard, chemotherapy-induced mitochondrial dysfunction and paraneoplastic neurological syndromes (PNSs) further complicate long-term patient outcomes [134], which warrants future studies for the integration of neuroprotective strategies into SCLC management.

Despite progress in understanding SCLC subtypes, challenges remain in translating these findings into effective clinical strategies. Tumor heterogeneity, biomarker limitations, and therapy resistance continue to hinder advancements, highlighting the need for innovative patient stratification and treatment optimization. The integration of epigenetic modulators, metabolic inhibitors, and immune-based therapies may improve treatment durability, while multi-omic data and real-time liquid biopsy monitoring will refine precision medicine approaches. By leveraging these cutting-edge technologies, the future of SCLC treatment will be more targeted, adaptable, and personalized, ultimately improving patient survival and outcomes.

## Figures and Tables

**Table 1:** Summary of upstream regulatory mechanisms of lineage-defining transcription factors that functionally impact different SCLC subtypes.

Transcription Factor	Regulator	Outcomes	Potential Treatments	Reference
ASCL1	TCF3/CDK2/Cyclin A2	Stabilization	CDK2 inhibitors	[53]
	HUWE1	Degradation	None	[52]
	USP8	Stabilization	USP8 inhibitors	[54]
NEUROD1	SMARCA4	Promotes its transcription	SMARCA4 inhibitors	[55]
	EZH2	Suppresses its expression	None	[62]
	LSD1	Enhances transcriptional activity	LSD1 inhibitor	[57]
	FMRP	Inhibits its translation	None	[58]
POU2F3	IGF1R	Promotes its expression	IGF1R inhibitors	[29]
	OCA-T1/ OCA-T2	Activation	None	[30]
	mSWI/SNF complex	Promotes its transcription	SMARCA2/4 inhibitor; BRD9 degrader	[59,60,61]

**Table 2:** Targeted therapeutic strategies for respective SCLC subtypes.

SCLC Subtype	Targeted Therapies
SCLC-A	BCL2 inhibitors (venetoclax), DLL3-targeting agents (tarlatamab), LSD1 inhibitors (ORY-1001), HDAC inhibitors (pracinostat), ATR inhibitors, PARP inhibitors (olaparib), CEACAM5-targeting agent (labetuzumab govitecan), Lurbinectedin
SCLC-N	MYC-targeting agents (OMO-103), AURKA/B inhibitors (alisertib, barasertib), IMPDH inhibitors, mTOR inhibitors, CHK1 inhibitors, BET inhibitors (CPI-0610, JQ1), SSTR2-targeting agents
SCLC-P	IGF1R inhibitors (linsitinib), PARP inhibitors, antifolate agents, SWI/SNF ATPase inhibitors (SMARCA4/2 inhibitors, BRD9 degraders)
SCLC-I	Immune checkpoint inhibitors (ICIs), BTK inhibitors (ibrutinib), HDAC inhibitors (pracinostat)
YAP1 Activation	RTK inhibitors (EGFR, FGFR), BTK inhibitors (ibrutinib)
RB1-Intact	CDK4/6 inhibitors (palbociclib, abemaciclib)
NMF Clusters	NMF1: PARP/ATR inhibitors; NMF2: DLL3 ADCs, ICIs; NMF3: RTK inhibitors; NMF4: AURKA inhibitors

**Table 3:** Challenges in targeting SCLC subtypes.

Challenge	Description	Potential Solutions
Tumor Heterogeneity	Coexistence of multiple subtypes within the same tumor	Single-cell RNA sequencing, spatial transcriptomics
Subtype Plasticity	Dynamic transition between subtypes under therapy pressure	Real-time monitoring with liquid biopsy
Resistance Mechanisms	Epigenetic reprogramming, clonal evolution, drug resistance	Combination therapy, synthetic lethality approaches
Limited Biomarkers	Lack of standardized predictive biomarkers for treatment selection	Integration of ctDNA, RNA-seq, and proteomics

## Data Availability

No new data were created or analyzed in this study. Data sharing is not applicable to this article.
